# Anterior vertebral body tethering for adolescent idiopathic scoliosis associated with less early post-operative pain and shorter recovery compared with fusion

**DOI:** 10.1007/s43390-023-00661-6

**Published:** 2023-02-21

**Authors:** Jennifer M. O’Donnell, Alex L. Gornitzky, Hao-Hua Wu, Kira S. Furie, Mohammad Diab

**Affiliations:** 1 Department of Orthopaedic Surgery, University of California San Francisco, 505 Parnassus Ave MU 320W, San Francisco, CA 94143 USA; 2 University of California San Francisco School of Medicine, San Francisco, CA 94143 USA

**Keywords:** Adolescent idiopathic scoliosis, Anterior vertebral body tethering, Patient-reported outcomes, Pediatric spine

## Abstract

**Purpose:**

While posterior spinal instrumentation and fusion (PSIF) for severe adolescent idiopathic scoliosis (AIS) is the gold standard, anterior vertebral body tethering (AVBT) is becoming an alternative for select cases. Several studies have compared technical outcomes for these two procedures, but no studies have compared post-operative pain and recovery.

**Methods:**

In this prospective cohort, we evaluated patients who underwent AVBT or PSIF for AIS for a period of 6 weeks after operation. Pre-operative curve data were obtained from the medical record. Post-operative pain and recovery were evaluated with pain scores, pain confidence scores, PROMIS scores for pain behavior, interference, and mobility, and functional milestones of opiate use, independence in activities of daily living (ADLs), and sleeping.

**Results:**

The cohort included 9 patients who underwent AVBT and 22 who underwent PSIF, with a mean age of 13.7 years, 90% girls, and 77.4% white. The AVBT patients were younger (*p* = 0.03) and had fewer instrumented levels (*p* = 0.03). Results were significant for decreased pain scores at 2 and 6 weeks after operation (*p* = 0.004, and 0.030), decreased PROMIS pain behavior at all time points (*p* = 0.024, 0.049, and 0.001), decreased pain interference at 2 and 6 weeks post-operative (*p* = 0.012 and 0.009), increased PROMIS mobility scores at all time points (*p* = 0.036, 0.038, and 0.018), and faster time to functional milestones of weaning opiates, independence in ADLs, and sleep (*p* = 0.024, 0.049, and 0.001).

**Conclusion:**

In this prospective cohort study, the early recovery period following AVBT for AIS is characterized by less pain, increased mobility, and faster recovery of functional milestones, compared with PSIF.

**Level of evidence:**

IV.

## Introduction

Adolescent idiopathic scoliosis (AIS) affects 1–3% of U.S. adolescents [1, 2]. Although posterior spinal instrumentation and fusion (PSIF) is the gold standard treatment, the resultant loss of spinal motion alters present function and may lead to long-term degenerative disc disease [3–8]. In contrast, emerging evidence supports the safety and efficacy of anterior vertebral body tethering (AVBT) as a novel technique that conserves motion and may permit growth modulation for spontaneous curve correction [9–11].

A recent meta-analysis comparing AVBT with PSIF found similar clinical outcomes, deformity correction, and mid-term Scoliosis Research Society-22 scores (SRS-22) [12]. However, revision and complication rates for AVBT were higher (14.1% vs. 0.6% and 26% vs. 2%, respectively). While multiple studies have examined health-related quality of life (HRQOL) following AVBT, there are no published prospective comparisons of AVBT versus PSIF with respect to post-operative analgesia and functional recovery [13–15].

The purpose of this study is to compare post-operative pain and functional recovery in AVBT versus PSIF using a prospective series of patients. We hypothesized that AVBT patients have significantly reduced post-operative pain and improved functional recovery up to six weeks after surgery because the procedure is less morbid to the osseous spine.

## Materials and methods

This was a prospective observational study at a single academic institution. Institutional review board approval was obtained prior to commencement. Consecutive children aged 11–18 treated with primary multilevel PSIF or AVBT by a single senior surgeon for idiopathic scoliosis from May 2019 through April 2021 were eligible. Exclusion criteria included non-idiopathic scoliosis, history of chronic pain/opioid use, history of prior spine surgery, non-English-speaking, and unplanned re-admission or return to the operating room within six weeks of index procedure.

Pre-surgical clinical data were obtained from the medical record. Cobb angles for curve magnitude were measured, and Risser scores were assessed when available from PA radiographs [16–18]. Clinical outcomes were assessed for the first six weeks after operation. Daily pain scores were collected for the first four weeks, and weekly thereafter. Opioid consumption and pain self-efficacy were queried weekly. The NIH’s Patient Reported-Outcomes Measurement Information System (PROMIS^®^) pain-related tools (Pain Behavior and Pain Interference) were measured at 1, 2 and 6 weeks [19]. These scales are well validated across a variety of orthopedic populations, compare well with legacy measures, and have a mean score of 50 with a minimum clinically significant difference of 3 [19–21]. Global satisfaction with pain management and overall treatment was gauged at 2 and 6 weeks. Time to key milestone completion (such as independent completion of activities of daily living (ADL) without assistance and sleeping through the night without waking for analgesics) was evaluated weekly. Ambulatory capacity (PROMIS Mobility) was assessed at 1, 2 and 6 weeks. Psychosocial health (PROMIS Anxiety, PROMIS Positive Affect) and health-related quality of life (HRQOL) (PROMIS Global) were measured at 2 and 6 weeks after operation. Each PROMIS measure was also collected before operation to provide an individual baseline. All PROMIS scores were measured by computer adaptive testing.

For inclusion in either the 2- or 6-week analysis, patients had to meet two of the following three criteria defined prior to study initiation: (1) completion of at least 50% of all surveys; (2) completion of at least 50% of the weekly check-ins; (3) completion of either the 2- or 6-week PROMIS measures (respectively). This minimum completion percentage was established in order to ensure the patients were appropriately engaged with the surveys and providing accurate responses. Patients have been followed for a minimum of 2 years after surgery.

Descriptive statistics were generated for demographic variables. Continuous variables were then analyzed using the student *t* test or the Mann–Whitney *U* test. Paired analyses were completed to compare change in outcome measures over time. Categorical variables were compared using the Chi-square tests. Categorical variables are reported as frequency and percentage; continuous variables are presented with a measure of central tendency (mean or median) and spread (SD or range). All comparative analyses were two tailed with alpha set at 0.05.

## Results

**Fig. 1:**
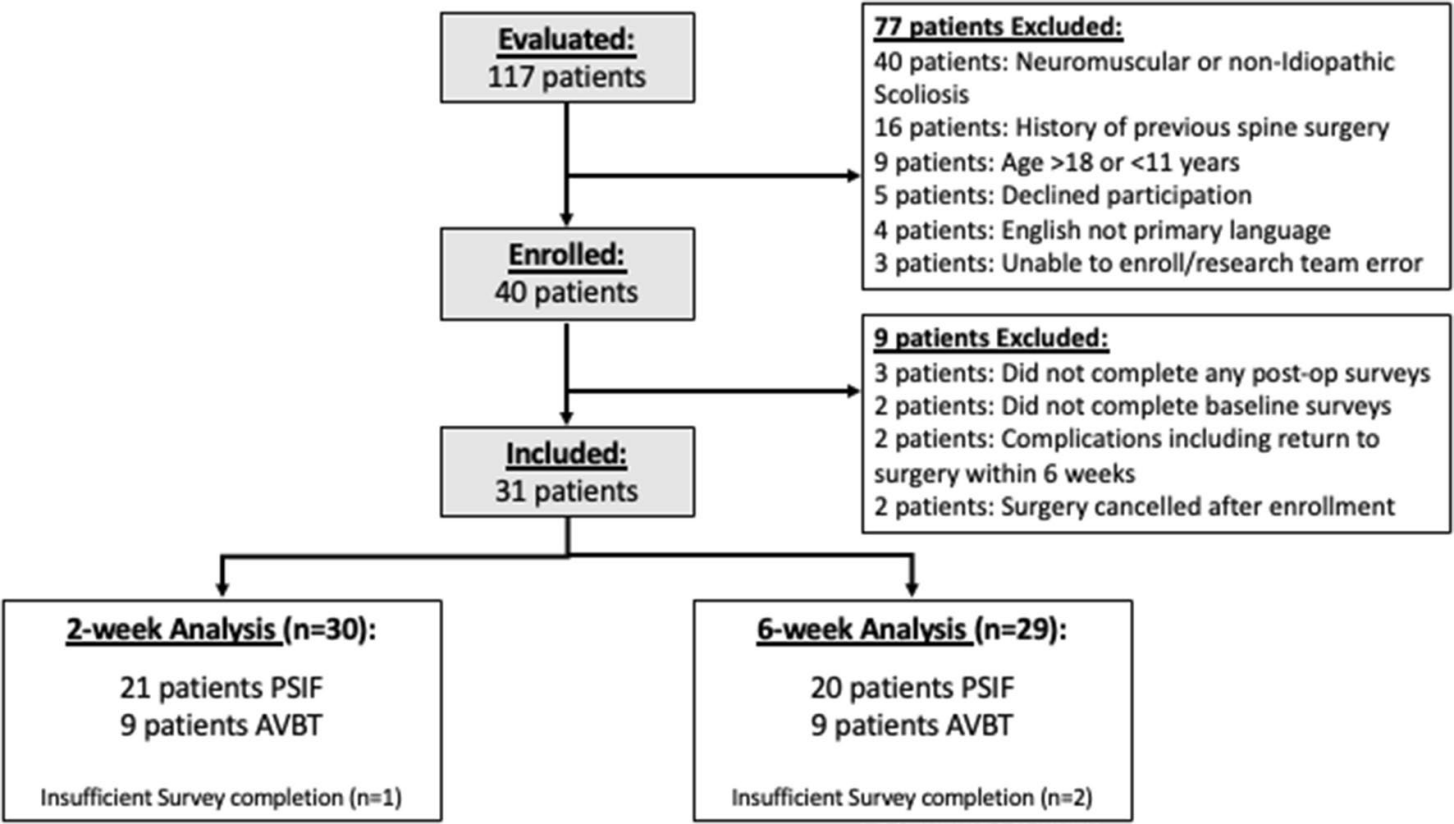
CONSORT diagram of recruitment. All surgical patients were screened for participation. All eligible patients were invited to join the study

Forty patients were enrolled. Nine patients were excluded after enrollment, including 5 who not complete the required surveys, 2 who canceled their surgery, and 2 patients who were excluded for return to surgery within 6 weeks. The two patients who had complications requiring return to surgery had undergone fusion surgery. There were 31 patients in the final cohort, 9 in the AVBT group and 22 in the PSIF group (Fig. 1). Mean age was 13.7 years, 90% (28/31) were girls and 77.4% were non-Hispanic white (Table 1). Those who underwent AVBT were younger (12.8 vs. 14.1 years) (*p* = 0.03). The AVBT patients were also less skeletally mature, with lower Risser scores (2.1 vs. 3.5 out of 5) (*p* = 0.03). There was no difference among the AVBT and PSIF groups in terms of gender (*p* = 0.26) or ethnicity (*p* = 0.29). The mean magnitude of the major curves were 54.5 degrees. Pre-surgical curve magnitudes of those who underwent AVBT were significantly less (47.7 vs. 57.5 degrees) (*p* < 0.001). Those in the AVBT group had a mean of 7.2 instrumented levels, which was significantly fewer than the mean of 9.7 in the PSIF group (*p* = 0.03).

**Table 1:** Demographics of fusion vs. tether groups

Characteristic	Total	Fusion	Tether	*p* value^a^
*n*	31	22	9	
Age	13.7 ± 1.6	14.1 ± 1.6	12.8 ± 1.2	**0.03**
Gender, % female (*n*)	90.3% (28)	86.4% (19)	100% (9)	0.26
Race, % (*n*)				
White	61.3% (19)	54.5% (12)	77.8% (7)	0.24
Black	3.2% (1)	4.5% (1)	0.0 (0)	
Latinx	12.9% (4)	13.6% (3)	11.1% (1)	
Ethnicity, % non-Hispanic	77.4% (24)	72.7% (16)	88.8% (8)	0.29
School Grade	8.5 ± 1.6	9.0 ± 1.6	8.2 ± 1.4	**0.03**
Risser score	3.2 ± 1.2	3.5 ± 1.6	2.1 ± 0.9	**0.03**
Curve magnitude	54.5 ± 7.0	57.5 ± 5.6	47.7 ± 5.0	** < 0.01**
Levels involved	9.0 ± 2.9	9.7 ± 2.9	7.2 ± 2.4	
Most common UIV^b^		T3 (14)	T5 (7)	**0.03**
Most common LIV		L4 (10)	T12 (4)	

Bold values signify *p* < 0.05

^a^Two-tailed *t* test was performed

^b^UIV, uppermost instrumented vertebra; LIV, lowest instrumented vertebra

**Fig. 2:**
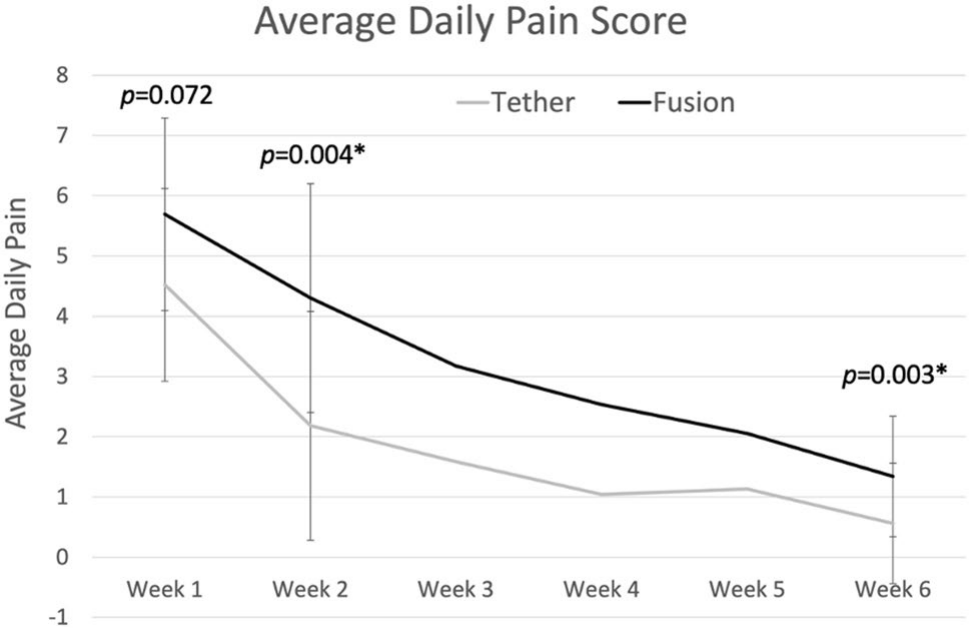
Average daily pain score following surgery. Patient’s response to the prompt: “On a scale of 0–10, with 0 being no pain and 10 being the worst pain imaginable, which one of the following best describes the average amount of pain you have experienced over the past day while doing activity (for example walking)?” Pain scores were collected daily for the first 2 weeks (and averaged), and then weekly thereafter

**Fig. 3:**
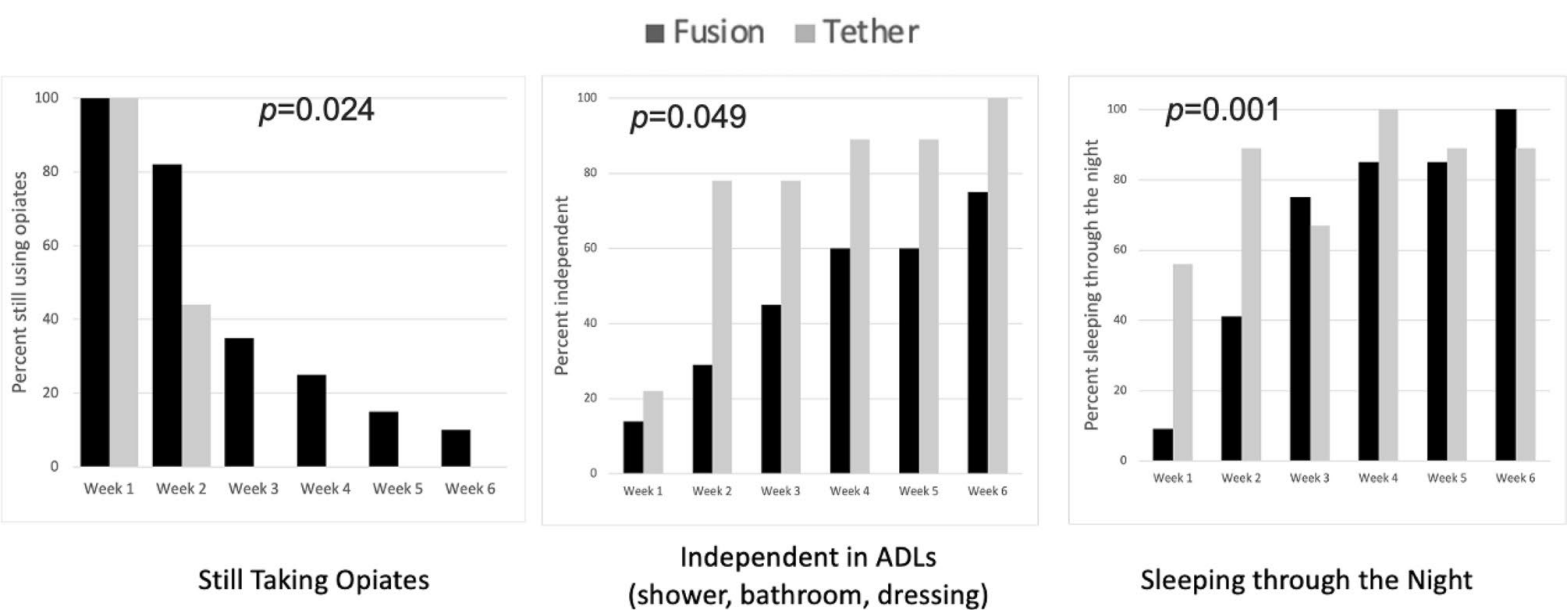
Confidence in ability to manage pain following surgery. Patient’s response to the prompt: “Based upon your experiences over the past week, how confident are you in your ability to control the level of pain you are having?” Responses were assessed weekly. Each bar is depicted with the *p *value for the associated comparative analysis between groups

Mean daily pain scores were significantly lower in those who underwent AVBT at both 2 weeks (2.2 vs. 4.3, *p* = 0.004) and 6 weeks post-operatively (0.6 vs. 1.3, *p* = 0.030) (Table 2, Fig. 2). There were no significant differences between groups in regards to their confidence in their ability to manage their pain, at any timepoint (Fig. 3).

**Table 2:** Outcomes comparison of fusion vs. tether groups

Characteristic	Fusion	Tether	*p* value
*n*	22	9	
Average daily pain score (0–10)			
1 week	5.7 ± 1.6	4.5 ± 1.4	0.072
2 weeks	4.3 ± 1.9	2.2 ± 0.6	**0.004**
6 weeks	1.3 ± 1.0	0.6 ± 0.5	**0.030**
Pain confidence (1–5)			
1 week	3.4 ± 1.1	3.9 ± 1.0	0.252
2 weeks	3.6 ± 1.0	4.3 ± 0.5	0.057
6 weeks	4.3 ± 0.8	4.8 ± 0.3	0.084
Functional milestones (weeks)			
Weaned off opiates	2.8 ± 1.6	1.4 ± 0.5	**0.024**
Independent with ADLs^a^	4.0 ± 1.9	2.4 ± 1.6	**0.049**
Sleeping through the night	2.7 ± 1.0	1.4 ± 0.5	**0.001**
PROMIS pain interference			
1 week	64.3 ± 7.9	59.3 ± 5.3	0.059
2 weeks	60.0 ± 6.2	53.7 ± 2.8	**0.005**
6 weeks	49.1 ± 6.2	41.7 ± 6.9	**0.007**
PROMIS pain behavior			
1 week	58.5 ± 5.0	54.6 ± 4.6	**0.032**
2 weeks	56.1 ± 4.6	49.4 ± 6.0	**0.001**
6 weeks	47.7 ± 8.1	34.5 ± 10.8	**0.002**
PROMIS mobility			
1 week	30.8 ± 3.5	33.9 ± 2.9	**0.026**
2 weeks	32.6 ± 4.1	36.4 ± 3.9	**0.019**
6 weeks	37.0 ± 4.	42.6 ± 7.2	**0.001**

Bold values signify *p* < 0.05

^a^ADL, activities of daily living

**Fig. 4:**
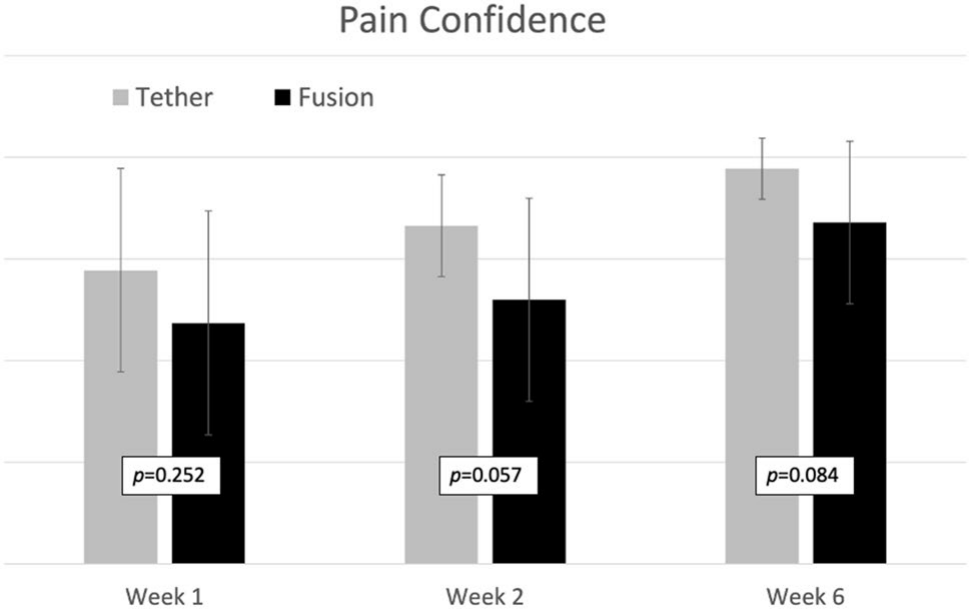
Completion of key milestones following surgery. Percent of patients in each group meeting each milestone in the weeks after operation. Responses were at the end of each week

Patients treated with AVBT achieved all functional milestones earlier than the PSIF group (Table 2, Fig. 4). They were quicker to both independent completion of ADLs (mean 2.4 weeks post-operatively versus mean 4.0 weeks post-operatively, *p* = 0.049) and sleeping through the night without waking in pain (mean 1.4 weeks versus mean 2.7 weeks, *p* = 0.001). Specifically, all AVBT patients were independent with ADLs by week 6 after operation compared with 25% of PSIF patients who remained dependent at this time point. Those in the PSIF group took twice as long to wean off opioids (mean 2.8 weeks post-operatively versus mean 1.4 weeks post-operatively; *p* = 0.024). Specifically, all AVBT patients were off opioids by week 3, whereas 10% (*n* = 2/19) of PSIF patients still were taking opioids at 6 weeks after operation (Fig. 4).

**Fig. 5:**
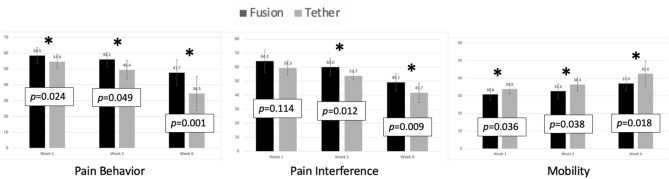
Post-operative PROMIS scores. Higher PROMIS scores represent more of the category being measured. Each bar is depicted with the specific PROMIS score as well as the *p* value for the associated comparative analysis between groups

PROMIS scores were significantly lower in the AVBT group across each of the primary outcomes of pain interference and pain behavior, and significantly higher for mobility (Table 2, Fig. 5). PROMIS pain behavior scores were significantly lower among AVBT patients at both 2 weeks (49.4 vs. 56.1, *p* = 0.004) and 6 weeks (34.5 vs. 47.7, *p* = 0.002). PROMIS pain interference scores were also lower at both 2 weeks (53.7 vs. 60.0, *p* = 0.012) and 6 weeks (41.7 vs. 49.1, *p* = 0.009). PROMIS mobility scores were significantly higher among AVBT patients as compared with PSIF patients at 1 week (33.9 vs. 30.8, *p* = 0.036), 2 weeks (36.4 vs. 32.6, *p* = 0.038) and 6 weeks (42.6 vs. 37.0, *p* = 0.018), with higher scores representing greater mobility. Each of these differences also met the minimum clinically significant difference. There were no between-group differences in any of the additional measured outcomes at either 2 weeks or 6 weeks timepoint, including PROMIS global health, anxiety, positive affect, physical activity or strength impact (all *p* > 0.05).

## Discussion

AVBT is an emerging treatment option for select patients with AIS. To date, there have been few prospective studies directly comparing patient-reported outcomes for patients undergoing AVBT versus those treated with PSIF. Our data show that AVBT patients have less pain and recover more quickly compared with PSIF patients. Specifically, those treated with AVBT had lower mean daily pain scores, faster time to discontinuation of opioids, earlier independent completion of ADLs, and less disturbances in sleep for the first six weeks after surgery. These findings were reflected across a number of validated patient-reported outcome scores, including lower PROMIS pain behavior and pain interference scores, and higher PROMIS mobility scores at 2 and 6 weeks post-operatively for AVBT patients. Notably, each of these findings surpassed the minimum clinically significant difference of 3 points for each pediatric PROMIS measure, further highlighting the value of this difference to patients and their families [20, 21].

We chose a follow-up period of 6 weeks because this time period encompasses opioid use, highest pain and greatest disability.

Our findings are consistent with prior reports that suggest long-term patient-reported outcomes for AVBT are as good as or better than those for PSIF [13, 22]. Qiu et al. found equivalent HRQOL scores in a retrospective comparison of 20 AVBT and 62 PSIF patients [13]. Additionally, in a comparison of 21 AVBT to 22 PSIF patients, Pehlivanoglu and colleagues found AVBT to have better SRS-22 and SF-36 MFS/PCS scores, which include measures for pain control, compared with PSIF at final minimum 2-year follow-up [22]. Here, our data confirm that AVBT patients had decreased pain compared with PSIF patients between two- and six weeks after operation. Additionally, our study identified a number of new findings not previously described, including earlier achievement of functional milestones such as time to opioid cessation and ability to sleep through the night without waking for pain.

Pain is the predominant concern for patients undergoing AIS surgery, as well as their parents [23, 24]. Higher patient and parental anxiety can have deleterious effects on outcomes, including increased opioid use [25]. The current study provides a predictive template for which patients, parents and surgeons can manage expectations for pain and function in the short-term following operation. For example, the ability to counsel patients on mean time to opioid discontinuation or what a standard pain trajectory looks like may help to alleviate pre-operative anxiety as families plan their post-operative recovery [26]. In fact, actual and expected pain trajectories often follow the same pattern [27]. Furthermore, there is a strong desire for additional pain-related information prior to surgery, as the post-operative recovery period can be very stressful for families [28, 29].

The principal limitation of our study is the small number of patients. This was due to the strict criteria for inclusion, in particular completion of surveys, which we also regard as a strength. In addition, the scope of our enrollment period was one year, which limits the number of patients who participated. We narrowed the time window to limit the impact of evolution of surgical technique for a novel procedure. Furthermore, a new procedure must be applied judiciously with strict inclusion criteria while long-term outcomes remain unknown; hence the small number of AVBT patients. Despite the small numbers, to our knowledge, this is the largest prospective study comparing perioperative pain between PSIF and AVBT. Additionally, here are different surgical indications for these two procedures, and as such, this comparison must be utilized and interpreted judiciously.

Using prospectively collected data from a single-surgeon cohort, and applying validated outcome measures, our findings aid decision-making for the novel technique of AVBT, focusing on recovery in the early post-operative period. Although a small cohort, this study can add information to the growing literature on the equipoise in treatment recommendations between AVBT and PSIF [30].

## Conclusion

Patients treated with AVBT had less pain in the early post-operative period compared with those who underwent PSIF. AVBT patients were also quicker to achieve every measured functional milestone in the first 6 weeks post-operatively, and had improved PROMIS scores for pain interference, pain behavior and mobility. It must be noted that the indications for AVBT are more narrow than PSIF. These data can enhance decision-making regarding this novel procedure, AVBT, for which clinical equipoise remains.

## Data Availability

All deidentified data is available upon request.
